# Acquiring and developing healthcare leaders’ political skills: an interview study with healthcare leaders

**DOI:** 10.1136/leader-2022-000617

**Published:** 2022-05-23

**Authors:** Justin Waring, Simon Bishop, Jenelle Clarke, Mark Exworthy, Naomi J Fulop, Jean Hartley, Angus I G Ramsay, Georgia Black, Bridget Roe

**Affiliations:** 1 Health Services Management Centre, University of Birmingham, Birmingham, UK; 2 Centre for Health Innovation, Leadership and Learning, University of Nottingham, Nottingham, UK; 3 Applied Health Research, University College London, London, UK; 4 Business School, The Open University, Milton Keynes, UK

**Keywords:** clinical leadership, politics, development

## Abstract

**Background:**

Research suggests health and care leaders need to develop a distinct set of political skills in order to understand and manage the competing interests and agenda that characterise health and care services.

**Aim:**

To understand how healthcare leaders describe the acquisition and development of political skills with the aim of providing evidence for leadership development programme.

**Methods:**

A qualitative interview study was carried out between 2018 and 2019 with 66 health and care leaders located within the English National Health Service. Qualitative data were subject to interpretative analysis and coding, with themes related to pre-existing literature on the methods of leadership skill development.

**Results:**

The primary method of acquiring and developing political skill is through direct experience in leading and changing services. This is unstructured and incremental in nature with skill development increased through the accumulation of experience. Many participants described mentoring as an important source of political skill development, especially for reflecting on first-hand experiences, understanding the local environment and fine-tuning strategies. A number of participants describe formal learning opportunities as giving them permission to discuss political issues, and providing frameworks for conceptual understanding of organisational politics. Overall, no one approach appears to reflect the changing developmental needs of leaders.

**Conclusions:**

The study suggests that healthcare leaders’ development of political skills and behaviours might be supported through an integrative approach that takes into account the evolving learning needs and opportunities at different career stages in the form of a maturation framework.

**WHAT IS ALREADY KNOWN ON THIS TOPIC**

- Healthcare organisations are complex political arenas and healthcare leaders need to use political skills when implementing change, but the process by which leaders in healthcare develop political skills has not yet been studied.

**WHAT THIS STUDY ADDS**

- This major qualitative study finds the acquisition of political skills among healthcare leaders occurs through a combination of training, mentoring and experiential learning, but none is sufficient alone.

**HOW THIS STUDY MIGHT AFFECT RESEARCH, PRACTICE AND/OR POLICY**

- Leadership development activities should include consideration of organisational politics and political skills and consider how elements of political skill can be matured through integrative learning methods at appropriate points in leaders’ careers.

## Introduction

 Leading in healthcare organisations involves dealing with the diverse agendas and interests of healthcare workers. Incompatibilities between these can lead to disagreement in the form of ‘micropolitical’ behaviours.^1^,^3^ Such political behaviour can be seen when healthcare professionals resist organisational changes that are perceived as threatening established ways of working,^4^ or otherwise seek to influence the organisation of work in line with individual or collective interests.^5^ Research suggests it is important for clinical leaders to understand the lines of power and influence in the healthcare workplace.^6 7^ Power, broadly defined, can be seen as concerned with the distribution of resources and rewards and how this affords variable degrees of influence or authority, with previous research identifying considerable complexities of power hierarchies in healthcare.^8^ It has therefore been suggested that those implementing change need to be ‘politically credible leaders’ who broker between competing interest groups, secure stakeholder buy-in and manage the political challenge of change.^9^ There has been some recognition of the link between politics and leadership within the National Health Service (NHS). The NHS Leadership Academy Healthcare Leadership Model, which presents desired leadership behaviours, explicitly states that leaders need to understand the culture and politics of their organisation, including the informal chain of command, with higher level leadership skills associated with expanding this understanding to the system level.^10^ Similarly, the Institute for Healthcare Improvement’s leadership development programme talks of the need to map stakeholders and resource and to leverage existing assets towards change.^11^ At the same time, questions of politics and power often remain implicit within many healthcare leadership development programmes or subsumed within frameworks for change management.

The strategic management literature has long suggested that strategic leaders need to develop and use political skills when implementing change.^12 13^ One prominent conceptualisation defines political skill as ‘(t)he ability to effectively understand others at work, and to use this understanding to influence others to act in ways that enhance one’s personal and/or organisational objectives’.^14^ Political skill has been examined in the healthcare context where it is shown, for example, that politically astute leaders can be more effective at introducing change, supporting team performance and managing conflict.^6^ In their recent review, Clarke *et al*^1^ offer a context-specific conceptualisation of healthcare leaders’ political skills in terms of personal performance (self-belief, self-efficacy, resilience), contextual awareness (understanding lines of power, interests of others, viable options), interpersonal influence (negotiating, persuading), stakeholder engagement and networking (communication strategies to mobilise and connect people) and influence of policy processes (influence on formal decision-making).

A key question for healthcare leadership is how such political skills can be acquired and developed.^3 6 15^ Within the leadership development literature, such skills, competencies and behaviours are usually acquired through a combination of three forms of learning.^16 17^ Formal training through class-room instruction, action learning and peer-based activities can be useful for introducing and relating frameworks on organisational politics to the ‘real world’ context of leadership.^18^ Mentoring and coaching can support leaders to reflect on first-hand experiences and impart knowledge about the local ‘rules of the game’.^19^,^22^ Experiential learning in the context of making decisions, facing opposition and taking actions offers a rich, but often haphazard mode of learning through making mistakes or through handling crises approach.^18 23^ Researchers also describe a maturation process where skills are ‘seasoned’ over time through direct participation in organisational politics.^19 20^ For example, Doldor^19^ describes the stages of ‘naivete and discovery’, ‘coping and endurance’ and ‘leveraging and proficiency’. Significantly, early positive experiences of using political skills are shown to have a positive impact of career development and subsequence maturation of skills and behaviours.^24^ It is also found that more senior organisational roles often present enhanced exposure to and opportunities to engage in organisational politics.^25^

Recent systematic reviews of leadership development literature level have suggested that combining educational methods may lead to improvements across a range of individual and organisational outcomes.^26 27^ However, there is little research investigating how healthcare leaders themselves describe the acquisition and development of political skills. Such insight could inform the future design of leadership development programmes inclusive of more formal programme, mentoring and experiential learning opportunities.

## Methods

### Design and sample

A qualitative interview study was carried out between 2018 and 2019 with people identified as leading the implementation of change in the English NHS. The interviews aimed to investigate the reflective experiences of acquiring, developing and using political skills with a focus on the relative contribution of different forms of training, mentoring and experiential learning.

Participants were purposefully sampled to maximise variation on criteria identified below.^28^ While participants were identified on the basis of their current role or position, it was also recognised that leadership is a dynamic, interactive and often distributed processes that goes beyond formal role designations. Furthermore, the interviews aimed to understand participants’ experiences of participating in change activities across their career, including those prior to their current role. Recruitment took into account differences across (1) career stage (early, middle and late), (2) leadership level and role (team, department, organisation, region, national), (3) care sector (primary, secondary, tertiary, community, mental health, social care) and (4) professional backgrounds (medical, nursing, allied, managerial).

Participants were identified and recruited through pre-existing connections within the English health and care system and snowball sampling of initial contacts.^29^ Over 80 names were identified and 50 were recruited through direct invitation. In addition, the study sampled people involved in formal leadership development programmes, who were recruited through a national network of NHS management trainees and sampling through university-based leadership programmes. This resulted in eight interview participants and a further eight people who participated in a focus group.

Of the 66 participants, 37 were female and 29 male; 59 were white British, 4 were Asian or British Asian, and 3 were black or black British. In terms of career background, the sample included: medical doctors (14), nursing and midwifery (18), allied health professionals (4), management (19), social care (3) and other (8), including charities, police services and care homes). Participants worked across a range of occupational roles, with many holding multiple roles, for example, in nursing leadership and quality improvement, from which 74 roles were identified (table 1). In terms of career experience, the sample was categorised into three groups: 10 people with less the 10 years, 23 people between 11 and 20 years, and 21 people with more than 30 years. It was not possible to determine the career length for 12 participants.

**Table 1:** Summary of interview participant’s occupational role

Role:	
Regional-level director	3
Quality/service improvement	18
External relations/communications	1
Local authority management	2
Primary care leadership	1
Medical leadership (hospital/regional)	5
Management (general)	17
Nursing leadership	6
Research leadership	2
Patient/public	3
Voluntary	5
Police leadership	1
Non-executive	3
National-level leader	3
National-level service improvement	4
Total	74

### Data collection and analysis

The interview study was designed to elicit participants’ experiences and understanding of political skill. Participants were asked to talk about prominent examples of change experienced during their career from which to reflect on their experiences of political skill and behaviour. Interview questions investigated how these skills have or could have been acquired and developed. Participants were also asked to reflect on their experiences of formal training, mentoring and workplace learning, together with recommendations for future leadership development. All interviews were recorded with the consent of participants and transcribed verbatim.

Interpretative data analysis^30 31^ started with all authors closely reading at least two transcripts to identify preliminary codes. Three members of the team (Bishop, Clarke, Waring) then systematically coded the data, with regular meetings to review the consistency of semantic coding and to undertake constant comparison to delineate latent themes. Interpretative analysis centred on participants’ experiences of the different methods and pedagogical approaches for acquiring and developing political skill and what these methods offered in terms of knowledge, skills and behaviours. The resulting analysis was related back to the existing literature on political skill, especially the work of Clarke *et al*^1^ with the intention of understanding how different approaches contribute to skill develop and for making recommendations for future leadership development. Consistent with the existing literature the findings are organised according to experiential learning, mentoring and coaching, and formal training (see online supplemental file for illustrative data and codes).

## Results

### Experiential learning

First-hand experience of leading teams and implementing change was the primary sources of learning about and developing political skills and behaviours. This learning was typically unstructured and based on direct involvement in a given change project (WP2-5) or taking on a formal leadership role. These experiential opportunities increased in frequency and intensity with length of career (WP2-27), with more experienced and senior participants describing a catalogue of learning opportunities that progressively enhanced their political skills. Most participants described a ‘seminal moment’ of ‘political awakening’ (WP2-31) which was often unsettling or akin to a ‘baptism of fire’ and involving some form of error of judgement or faux pas (WP2-63).

Certain care settings were described as being especially ‘rich’ in experiential learning, such as teaching hospitals where high-status specialists with established lines of power could routinely complicate change initiatives (WP2-37). Career moves between care settings could bring to light subtle differences in organisational politics. For a small number, exposure to more formal (big ‘P’) political processes in the form of secondments were an important source of learning about the political skills used by politicians in their day-to-day work (WP2-35).

Experiential learning was found to offer three common contributions to leaders’ political skill development. First, participants described how it heightened contextual awareness of the political landscape, especially the ability to read situations and understand competing agendas. Second, accumulated experience, especially negative experiences, was reported by participants as enhancing’ personal performance, particularly resilience with regards to conflict (WP2-4). Third, more experienced and senior-level participants described the cultivation and utilisation of different political strategies honed over the course of leading multiple change initiatives (WP2-5-D). This includes aspects of both inter-personal influence and stakeholder engagement and networking. Accounts highlighted how strategies, arguments or inducements had been successfully used to deal with certain professional groups, much of which seemed to operate as a form of tacit know-how.

### Mentoring

Mentors were described as helping participants to understand and navigate organisational politics. Some mentoring relationships were formally established as part of training or induction programmes, with the express purpose of supporting reflective learning in concert with a given development framework (WP2-35). This included, for example, helping to relate the materials or resources from formal training to the real-world challenges of leadership. More informal and opportunistic mentoring arrangements were usually based on long-standing (WP2-4) and trusted workplace relationships (WP2-1). Neither form of mentoring explicitly or exclusively dealt with organisational politics, rather they provided an open reflective space.

Mentoring relationships contribute to the development of political skills and behaviours in a number of ways. Assisting in the development of contextual awareness, mentors were seen to help participants interpret and conceptualise the local political landscape (WP2-35). Mentors were described as enhancing personal performance through providing a ‘safe space’ for reflection and to receive ‘constructive criticism’ (WP2-31), which could be especially important when trying to resolve difficult issues (WP2-35). Mentors were also seen as facilitating network and stakeholder connections, by making preliminary introduction on behalf of the individual or endorsing their involvement in a given activity (WP2-37). In related ways, mentors could provide a sense of security in a new or hostile environment.

### Formal training

Most participants had experienced some formal leadership or management training, including national training programmes, specialist clinical leadership programmes and short courses in leadership development. Despite such widespread experience, only a small number (fewer than 15 out of 66) described formal training in organisational politics or political skill (WP2-3). Some enrolled in graduate management training schemes described interactive sessions that introduced concepts related to organisational politics and ‘soft power’. Such training was widely described as raising awareness and giving participants permission to speak about workplace politics. However, some described a reluctance to talk about such issues in training sessions, favouring instead the opportunity to reflect on their heightened awareness through informal peer networks.

A small number discussed attending bespoke training workshops. These more directly addressed the practical demands of dealing with organisational politics and introduced different frameworks for appraising the local political landscape and developing strategic responses (WP2-47). Some memorable ideas, for example, were associated with Baddeley and James’s characterisation of ‘political animals’^32^ (WP2-47).

Formal education could therefore be seen as providing a structured space to engage with conceptual frameworks and to apply these frameworks in ways that allows for reflection on personal performance and political context (WP2-47). There was a general view that there is a ‘theory/practice’ gap within most training, for example, where participants found it difficult to relate concepts to real-world situations. This was especially the case for earlier career participants because they had insufficient first-hand experience from their work. For many, formal learning worked best when it could be taken into practice settings with the support of complementary mentoring and peer networks (WP2-35).

## Discussion

The findings suggest that in different, but only partial ways, formal training, mentoring and experiential learning are effective at supporting the acquisition and development of health and leaders’ political skills. Consistent with the wider literature,^19 24^ experiential learning supports leaders’ awareness and reading of the political context, personal resilience to cope with such politics and, over time, strategies and skills to influence the implementation of change. As found by Manzie and Hartley such experiential learning is typically unstructured and haphazard, with emotional costs,^18^ and echoing Oerder *et al*, those at more senior levels have greater exposure to such experiences through their increased involvement in change processes.^25^

Also supporting the existing literature,^19 21^ mentoring offers leaders a structured and reflective space for sense checking their understanding of the local political context and appraising their behaviours. Significantly, mentoring can supplement (and provide a link between) both experiential learning and more formal training. Mentoring arrangements cover a range of leadership matters beyond organisational politics, which might have the further advantage of integrating political with other leadership capabilities.^19^ It follows, however, that effective mentoring in political skills is dependent on the political skills of the mentor and their willingness of the mentee to discuss such issues.^24^

Consistent with the wider literature,^21^ formal training can raise awareness of organisational politics and introduce academic concepts and frameworks. More significant, and not well explored in the literature, is the potential for training courses to give leaders a sense of ‘permission’ to discuss political issues that might be regarded as taboo or covert in other areas of their work. However, formal training can be decoupled from the realities of day-to-day work, especially for those relatively early within their career with limited exposure to organisational politics.^19^

The immediate implication of the study is that it is unlikely any single learning method can provide the developmental support for healthcare leaders to acquire and develop political skills.^17^ Political skill development might, therefore, be supported through a combined or integrative approach^33^ that facilitates learning across multiple interconnected methods in a structured, rather than unplanned, way to facilitate real-world learning. This combined approach needs to link the evolving ‘real-world’ experiences that leaders accumulate over their career to relevant frameworks, but in ways that allows for reflective experimentation with the support of mentors. As such, there are clear advantages in designing relevant leadership development programmes that integrate the different methods of learning.^16^ This compliments the existing literature which identifies a combination of formal learning, mentoring and active methods as most likely to lead to improvements in individual and organisational outcomes.^26^ Furthermore, and linking the study results back to Clarke et al's framework on healthcare leaders’ political skill,^1^ it is possible to reflect on how the different forms of learning contribute to the development of particular skills in complementary ways and across the career trajectory. For example, experiential learning offers clear opportunities for developing contextual awareness, personal performance and networking abilities, but these can be enhanced through mentoring relationships that can open doors to different networks and support reflective learning, while formal learning can provide frameworks to more broadly analyse political dynamics in a structured and evidence-based way.

Also consistent with the wider literature,^19^ the study shows that healthcare leaders’ acquisition and development of political skills occurs progressively over the course of their career. As shown by Till and McGivern^34^ the ‘seeds of leadership development’ can take root early in the career and through progressive exposure to crucible moments, leadership competencies can be incrementally fine-tuned, often in concert with targeted development opportunities. This resembles a maturity model of political skill development where increasing length of service and career advancement affords greater exposure to and seasoning in organisational politics and, in turn, opportunities to hone political skills.^25^ In terms of healthcare leadership development, this suggests that training activities could be tailored to the developmental needs of leaders at different career stages with the goal of accelerating the maturation process. Early career leaders with limited direct exposure to organisational politics may benefit from more structured learning with case examples that provides a cognitive framework for orienting future experiences. Those with more first-hand experience can engage in more advanced formal learning where theory is applied to practice, and at the same time, these experiences provide the foundation for reflective peer learning and mentoring. As such the design of integrative learning should take account of the changes in exposure and experience over the career. Further, political skill can be extended and refined through taking on organisational roles especially those that are outside more traditional professional practice areas, in the form of hybrid professional-managerial roles. Such hybrids are likely to develop and use such skills as they mediate competing preferences^35^

The study has several limitations. First, it has not focused on a specific or particular learning scheme or programme and is not providing an appraisal of how such learning contributes to skill development. Second, the participants were recruited through existing research networks and there is therefore potential for bias in the views collected. Third, the study has not systematically measured participants’ acquisition or development political skill through an established survey and measure, and the findings are therefore subject to the interpretative biases of participants with regards to their own and others’ political skill. Furthermore, the research investigated participants own interpretative understanding of political skill rather than a prescribed framework and as such it does not focus on or operationalise a specific conceptualisation of political skill.

## Conclusions

Political skill acquisition is important for leaders’ ability to navigate the diverse and competing interests that complicate healthcare change. In contrast to other aspects of leadership development, political skills have received relatively little attention or are subsumed within other frameworks and models. The study finds that the acquisition and development of political skills is often haphazard, serendipitous and painful. The study recommends that healthcare leaders’ development of political skills and behaviours can be supported through an integrative approach that takes into account the evolving learning needs and opportunities at different career stages. It is recommended that relatively early career leaders be offered structured training sessions that raise awareness of organisational politics and encourage reflective conversations about such issues with peers and mentors. Initial training may also include opportunities for the vicarious experience of political issues, for example, by including case descriptions or direct accounts, with opportunity for critical reflection. More experienced leaders may benefit from structured training that offers advanced frameworks that help analyse and explain their acquired experiences and fine-tune their skills, again with support of mentors. Finally, those with more developed exposure to organisational politics and experience of engaging in political behaviour may benefit from problem-based or situational mentoring and action learning with peer networks to address the challenges of specific issues. As such, an integrative approach to continuous skills development is proposed.

## Supplementary material

10.1136/leader-2022-000617 online supplemental file 1

## Data Availability

Data are available on reasonable request.
