# Supplementary material for: Acquiring and developing healthcare leaders’ political skills: an interview study with healthcare leaders
Source: BMJ Lead. 2022 May 23;7(1):e000617. doi: 10.1136/leader-2022-000617 (PMC12038090; doi:10.1136/leader-2022-000617)
Supplement: online supplemental file 1 [file leader-7-1-s001.pdf]

.

Table 2: Coding and Illustrative data

Theme	Sub-Themes/Codes	Illustrative Data
Experiential Learning	Experience over time	'I think you get a bit of political nous with experience and it's about, I suppose it's about understanding well the internal politics they call it don't they, so it's about what … Because a lot of that I think is unspoken, you just learn it with experience and you learn to read between the lines a bit more. It's stuff that's not necessarily overt, but you pick up on that more discreet intelligence, soft intelligence should I say' [WP2-27]
	Learning from projects	'I remember … putting across a proposal that I thought was quite sensible and… the Paramedic Team Leader at the coffee break said you'll never get that through here because this is going on and you need to know the background here and actually it's something about understanding the politics of the [organisation] or that division, so what are those other things that are going on that, so that's part I think of being politically aware and politically astute I suppose internally.' [WP2-5]
	Accumulation experience over career	'...becoming the Deputy of this place [name] was a big step up for me and I suddenly found myself being the boss and I didn't know how to be that person to be honest and I, so actually learning to be myself in a different role was hugely challenging and the source of some considerable self-doubt…. I've, I think one of my strengths, and I've got many weaknesses, is I'm reasonably good at reading situations of people and that's a hugely helpful skill where one, to a degree, abstracts one's self from the heat and burden of the situation and you're very expert on what's going on here'. [WP2-31]
	Seminal moments	'…nobody prepares you for that and so that was a very seminal set of experiences for me and I think there's a correlation between the amount of pain one feels and the amount of leadership development one experiences.' [WP2-31]
	Mistakes	'Although we knew it was likely to be political, I don't think enough attention was given to the upfront analysis of what that might look like before we actually went to the first [project] meeting…[there were] sticking issues'. [WP2-63]
	Learning from formal political processes and other settings	'…[what] I found fascinating about working at a national level was that I really quite enjoyed some of the political, the working because it …I think it deepens your understanding about how systems work and how people work and how to get things done.' [WP2-35]

	Learning from other settings	'Being in different sectors, that in itself, opens up your mind to different ways of working and obviously the people within those sectors, have got different experiences.' [WP2-37]
	Context-specific understanding	'Be able to look at the wider picture … I did that when I was doing that job to go really wide and bring it down.' [WP2-4]
	Personal resilience	'I think you have to be resilient as well, you need to not take things personally which is quite a tension really because you're using a lot of personal skills to sell but then you can't take any negativity personally.' [WP2-4]
	Fine-tune skills	'…it's really important to be articulate, be assertive and I think what comes with experience, and I didn't have this when I started off as a Nurse Director, it just takes time to really understand others' perspectives because I think I was probably a bit bombastic …. I think that I needed to just moderate my approach a bit.' [WP2-5-D]
Mentoring and Coaching	Formal mentoring	'I went on the NHS management training scheme and as a result of that you get a mentor and you're also part of a learning set…' [WP2-35]
	Informal mentoring	'I've been really fortunate with mentors, unofficial mentors, official mentors, role models, [Name] was one of them. And some other key people that I've developed relationships with that have helped me and people that I've looked up to and seen how they work and tried to emulate that myself.' [WP2-4]
	Trusted colleagues	'I think just being able to reflect with someone that sort of you've built some level of trust with, who isn't a peer, because your peer is more likely to reinforce your behaviour and your culture as well.' [WP2-1]
	Reflection<br>Awareness	'…the opportunity to reflect with others on what I was experiencing in my roles and for them to reflect with me what they were experiencing, through conversations you're very aware of the politics and also thinking about how you act, what, how you conduct yourself and how you might act in a certain situation and I think when you have a mentor in your career, again that's someone who is able to give you a perspective on how they have managed organisational and individual politics and so I think I was aware of it as soon as I entered the world of work.' [WP2-35]
	Reflection	'But someone who's more senior to you, and more experienced than you, is able to challenge you, but also has experience, can reflect on their own experiences, and tell you things that you wouldn't have

		noticed before. That's really useful, that's really, really useful. And just observing people's behaviour in a situation.' [WP-2-1]
	Safe space	'I think really helpful to have a safe place and the important thing is safe where one can say look this is what I think is going on, have you got any ideas and then what you're doing is you're crowd sourcing amongst a small group of trusted mates.' [WP2-31]
	Support in the organisation	'…in my career, there are three people…that have actually influenced me, given me support when I've wobbled, when I've been fearful of standing up and doing what I think is the right thing, and standing my ground'.[WP2-37]
Formal training	Formal training	'… given some training on sort of organisational politics [Yeah] which is largely along the lines of 'organisational politics is a sin,' is a 'thing you have to deal with.' and it's sometimes a thing that can be used to, not necessarily to your advantage but to everyone's advantage. [WP2-2]<br><br>'We had a workshop, sort of day, where we talked about organisational politics, and we use some models. They sort of explained some models to us and did some interactive activity.' [WP2-3]
	Introduce theory and concepts	I can't remember the animals now, there was a *fox*, there was an *owl*, there was a *sheep*.… [what] was really powerful is that we have eighteen people as a cohort and you get to discuss politics and power openly, you can discuss the use of things and gives you the courage to go and test that. [WP2-47]
	Raising awareness<br>Giving permission	[we] had some kind of training in how to frame problems, change, help develop people's thinking in certain ways, but I really, it was really new to me and it made so much sense and it was, it was like a relief that somebody was naming power and politics. … It takes so much guilt away…it needs to have people who have got some ideas and some experience and some, and some, if you can call it theory, but at least some idea of the situation that requires the use of power and the use of politics. WP2-47
	Peer networks	I think the opportunity to reflect with others on what I was experiencing in my roles and for them to reflect with me what they were experiencing, through conversations you're very aware of the politics and also thinking about how you act, what, how you conduct yourself and how you might act in a certain situation]WP2-35]
